# *Ganoderma formosanum* polysaccharides attenuate Th2 inflammation and airway hyperresponsiveness in a murine model of allergic asthma

**DOI:** 10.1186/2193-1801-3-297

**Published:** 2014-06-12

**Authors:** Chia-Chen Pi, Hui-Yi Wang, Chiu-Ying Lu, Frank Leigh Lu, Chun-Jen Chen

**Affiliations:** Department of Biochemical Science and Technology, National Taiwan University, Taipei, 10617 Taiwan; Department of Pediatrics, National Taiwan University Hospital and National Taiwan University College of Medicine, Taipei, 10041 Taiwan

**Keywords:** Airway hyperresponsiveness, Allergic asthma, *Ganoderma formosanum*, Immunomodulation, Polysaccharides

## Abstract

Allergic asthma is an inflammatory disease of the airways mediated by Th2 immune responses and characterized by airway hyperresponsiveness (AHR). Fungi of the genus *Ganoderma* are basidiomycetes that have been used in traditional Asian medicine for centuries. We recently found that PS-F2, a polysaccharide fraction purified from the submerged culture broth of *Ganoderma formosanum*, stimulates the activation of dendritic cells and primes a T helper 1 (Th1)-polarized adaptive immune response. This study was designed to investigate whether the Th1 adjuvant properties of PS-F2 could suppress the development of allergic asthma in a mouse model. BALB/c mice were sensitized by repeated immunization with chicken ovalbumin (OVA) and alum, followed by intranasal challenge of OVA to induce acute asthma. PS-F2 administration during the course of OVA sensitization and challenge effectively prevented AHR development, OVA-specific IgE and IgG1 production, bronchial inflammation, and Th2 cytokine production. Our data indicate that PS-F2 has a potential to be used for the prevention of allergic asthma.

**Electronic supplementary material:**

The online version of this article (doi: 10.1186/2193-1801-3-297) contains supplementary material, which is available to authorized users.

## Background

Allergies or hypersensitivity is an immune disorder that occurs when the immune system reacts to noninfectious and normally innocuous environmental antigens (allergens). Allergens first stimulate/sensitize an adaptive immune response with the development of immunological memory in predisposed individuals. Subsequent exposures to allergens activate memory response, resulting in inflammation and tissue damage that can sometimes be fatal (Galli et al. 2008). Immunoglobulin E (IgE)-mediated allergic reaction results from the binding of allergens to allergen-specific IgE bound to its Fc receptor, primarily on mast cells. Crosslinking of Fcϵ receptors causes the degranulation of mast cells and the release of inflammatory mediators, which then recruit leukocytes from the blood. Both innate immune cells (monocytes, eosinophils, and neutrophils) and adaptive immune cells (T and B lymphocytes) are recruited to the site of allergen challenge. The recruited T lymphocytes are primarily CD4^+^ T helper (Th) cells secreting IL-4, IL-5, and IL-13; thus, IgE-mediated allergy is recognized as a Th2-skewed immune response (Galli et al. 2008).

In allergic asthma, inflammatory reactions occur in the lower airways and cause difficulties in breathing. Within seconds of mast-cell degranulation, fluid and mucus are secreted into the respiratory tract, and contraction of the smooth muscle surrounding the airway causes bronchial constriction. During the late-phase reaction, the Th2 cytokines produced by mast cells and T lymphocytes together induce changes in the airways and lung parenchyma. Repetitive or persistent exposure to allergens cause chronic inflammation of the airways with a persistent infiltration of leukocytes, resulting in epithelial injury, thickening of the airway walls, increased deposition of extracellular-matrix proteins, hyperplasia of goblet cells, and mucus hypersecretion (Hamid and Tulic 2009). The inflammatory and structural changes in the airways lead to airway hyperresponsiveness (AHR), which is a clinical feature of bronchial asthma and is closely associated with the severity of the disease. In chronic asthma, the airways are in a state of generalized hyperresponsiveness, and environmental factors other than reexposure to specific allergens can also trigger asthmatic attacks (Leikauf 2002).

Currently, patients with allergic asthma are primarily treated with inhaled corticosteroids and bronchodilators, and leukotriene receptor antagonists. Corticosteroids can modulate Th2 cytokine production and dampen the associated inflammatory responses. However, the effect of corticosteroids is broad and nonspecific; thus, therapeutic approaches with specific targets have also been developed (Barnes 2004). Allergy immunotherapies that aim to induce specific immune tolerance to allergens have been used in clinical practice for a century, and allergens delivered subcutaneously or sublingually have both been shown to prevent the development of asthma (Burks et al. 2013; Fitzhugh and Lockey 2011). Although effective in many patients, allergy immunotherapy is not successful in all individuals, and there remains the risk of allergen-induced anaphylaxis. More recently, new treatment strategies have been designed to target components of the Th2 pathway using biologics (Pelaia et al. 2012), such as IL-4Rα antagonists (Wenzel et al. 2007), and antibodies to IgE (Rodrigo et al. 2011), IL-13 (Ingram and Kraft 2012), and IL-5 (Walsh 2013). Accumulating evidence is supporting the efficacy of biological therapies in treating allergic asthma; however, current data also show that patients’ individual responses to these therapies are variable, highlighting the heterogeneity in asthma patients and the need to develop phenotype-targeted therapies (Pelaia et al. 2012). Besides blocking the effector molecules of the Th2 pathway, approaches targeting innate immunity have also been designed since the innate immune response can influence the development of Th subsets (Zhu et al. 2010). Activation of many TLRs results in IL-12 production by antigen-presenting cells (APCs), therefore skewing the cytokine balance from Th2 to Th1. Synthetic agonists for TLR4, TLR7, and TLR9 are currently studied in clinical trials for the treatment of asthma and allergies (Bezemer et al. 2012).

Medicinal mushrooms have been used as health-promoting supplements in Asia for centuries, and modern scientific research has revealed that the polysaccharides and proteins derived from mushrooms exhibit potent immunomodulatory activities (Li et al. 2011; Wasser 2002; Xu et al. 2011). The higher basidiomycete *Ganoderma* (also called Ling-Zhi or Reishi) is one of the most studied medicinal fungi, and various pharmacologically active constituents of *Ganoderma* have been characterized (Boh et al. 2007; Paterson 2006). *Ganoderma formosanum* is a native *Ganoderma* species isolated in Taiwan, and we have previously shown that a polysaccharide fraction, PS-F2, purified from the submerged culture fluid of *G. formosanum* stimulates macrophage activation by activating Toll-like receptor 4 (TLR4), Dectin-1, and complement receptor 3 (Wang et al. 2012; Wang et al. 2011). Furthermore, we recently showed that by stimulating the maturation of dendritic cells, PS-F2 could serve as a Th1 adjuvant and activate antitumor cytotoxic T cell responses (Pi et al. 2014). These observations led us to hypothesize that by stimulating a Th1-skewing immune response, PS-F2 could potentially suppress Th2-mediated allergic inflammation. In the present study, we tested this hypothesis and found that the administration of PS-F2 during the course of allergen sensitization and challenge could attenuate Th2 inflammation and AHR in a murine model of allergic asthma.

## Results

### PS-F2 treatment alleviates OVA-induced AHR in mice

**Figure 1:**
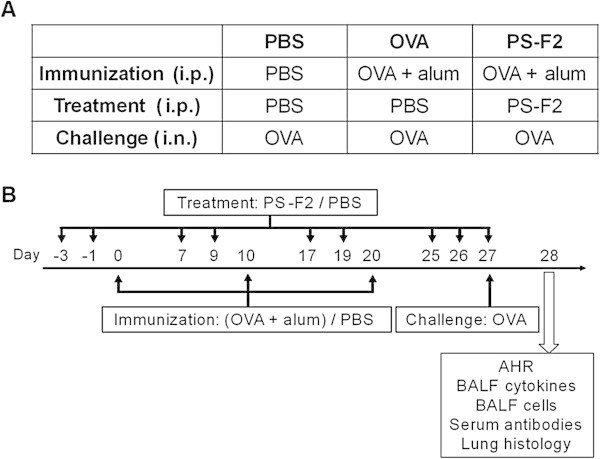
**Schematic diagrams of the experimental group design and protocol for chicken ovalbumin (OVA)-induced allergic asthma. (A)** Female BALB/c mice were divided into PBS, OVA, and PS-F2 groups (*n* = 10) which received different immunizations and treatments as indicated. **(B)** Mice were immunized with OVA + alum or PBS on days 0, 10, and 20. Mice also received treatment with PS-F2 or PBS on the indicated days. All animals were challenged i.n. with OVA on day 27. Twenty-four hours after OVA challenge, airway hyperresponsiveness (AHR) was measured, mice were sacrificed, and serum, bronchoalveolar lavage fluid (BALF), and lung tissue samples were collected for further analysis.

**Figure 2:**
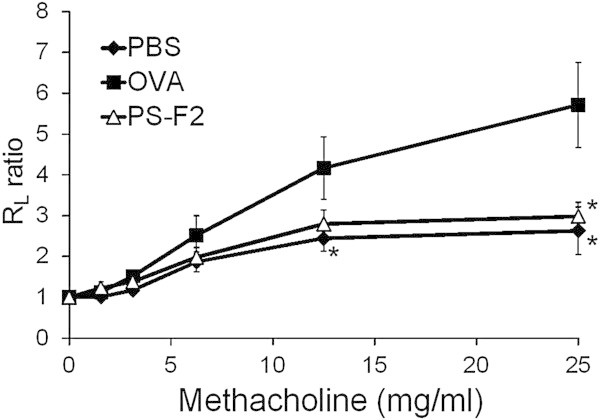
**PS-F2 treatment alleviates OVA-induced AHR in mice.** Mice were immunized, treated, and challenged as described in Figure 1. Airway responses to methacholine were measured with the flexiVent apparatus 24 h after i.n. OVA challenge. Data are presented as the ratio of the lung resistance (R_L_) at a given dose of methacholine compared to that obtained with PBS (*n* = 10). Data shown are representative of 2 experiments. *P < 0.05 versus OVA group.

To investigate whether PS-F2 could modulate a Th2-biased immune response, we examined the effect of PS-F2 treatment on OVA-induced allergic asthma in mice. Animals were divided into three groups (PBS, OVA, and PS-F2), which each received different treatments (Figure 1A). Allergic asthma was induced by first sensitizing mice with three i.p. immunizations with OVA + alum on days 0, 10, and 20, followed by an i.n. challenge of OVA on day 27 (Figure 1B). To evaluate the effect of PS-F2 on allergic asthma induction, mice were also treated i.p. with PBS or PS-F2 several times during the experimental period (Figure 1B). Twenty-four hours after the i.n. OVA challenge, AHR was measured with increased doses of methacholine by using the flexiVent system. As shown in Figure 2 and Additional file 1, mice immunized and challenged with OVA showed significantly increased AHR following methacholine exposure compared with the control animals (PBS group), which received only the i.n. OVA challenge but were not pre-sensitized by OVA immunization, indicating that the OVA-immunized/challenged mice developed symptoms of allergic asthma. In clear contrast to this, the AHR response was significantly attenuated in the OVA-immunized/challenged mice that had also been given PS-F2 (Figure 2 and Additional file 1), indicating that PS-F2 treatment suppressed the development of OVA-induced allergic asthma in mice.

### PS-F2 treatment attenuates bronchial inflammation in OVA-challenged mice

**Figure 3:**
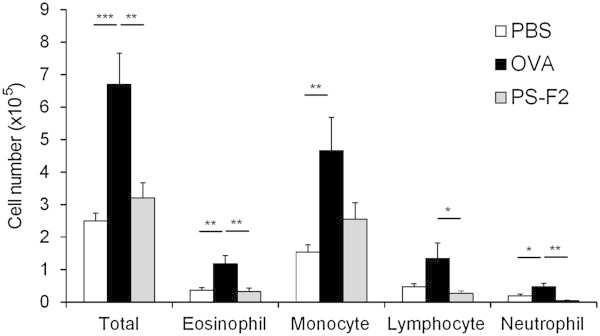
**PS-F2 treatment attenuates bronchial inflammation in OVA-challenged mice.** Mice were immunized, treated, and challenged as described in Figure 1. BALF was collected on day 28, and the numbers of total BALF cells, eosinophils, monocytes, lymphocytes, and neutrophils were determined by microscopic differential cell counts (*n* = 10). Data shown are representative of 2 experiments. * *P* < 0.05, ** *P* < 0.01, *** *P* < 0.001.

**Figure 4:**
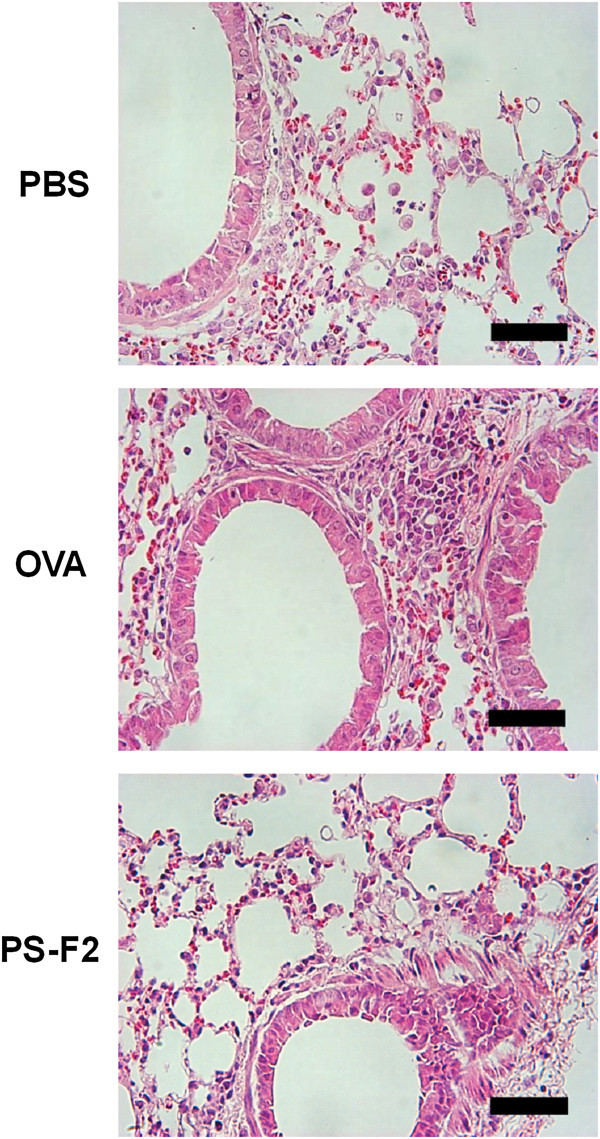
**PS-F2 treatment attenuates inflammatory cell infiltration of the airways.** Mice were immunized, treated, and challenged as described in Figure 1. On day 28, lung sections were prepared, stained with hematoxylin and eosin, and photographed under light microscopy at × 400 magnification (scale bar = 50 μm). Prominent infiltrates of inflammatory cells are present in OVA group mice but not in PBS and PS-F2 group mice.

Airway eosinophilic inflammation is a characteristic feature of asthma. To determine whether attenuated bronchial hyperresponsiveness in PS-F2-treated mice was associated with reduced airway inflammation, we also analyzed the recruitment of inflammatory cells into the airway walls by counting the cells in BALF and via histological examination of the lungs. Differential BALF cell counts revealed that OVA immunization and challenge resulted in a marked bronchial infiltration of inflammatory cells, including eosinophils, monocytes, lymphocytes, and neutrophils (Figure 3 and Additional file 2), whereas the recruitment of all types of inflammatory cells was significantly reduced in the PS-F2-treated animals (Figure 3 and Additional file 2). Extensive inflammatory infiltrates into the peribronchial areas were clearly seen in OVA-immunized/challenged mice by histological examination of lung tissue sections (Figure 4). In contrast, inflammatory infiltrates were markedly attenuated in mice treated with PS-F2 (Figure 4). These data indicate that treatment of animals with PS-F2 during OVA sensitization and challenge strongly suppressed OVA-induced airway inflammation.

### PS-F2 administration suppresses OVA-induced Th2 immune responses

**Figure 5:**
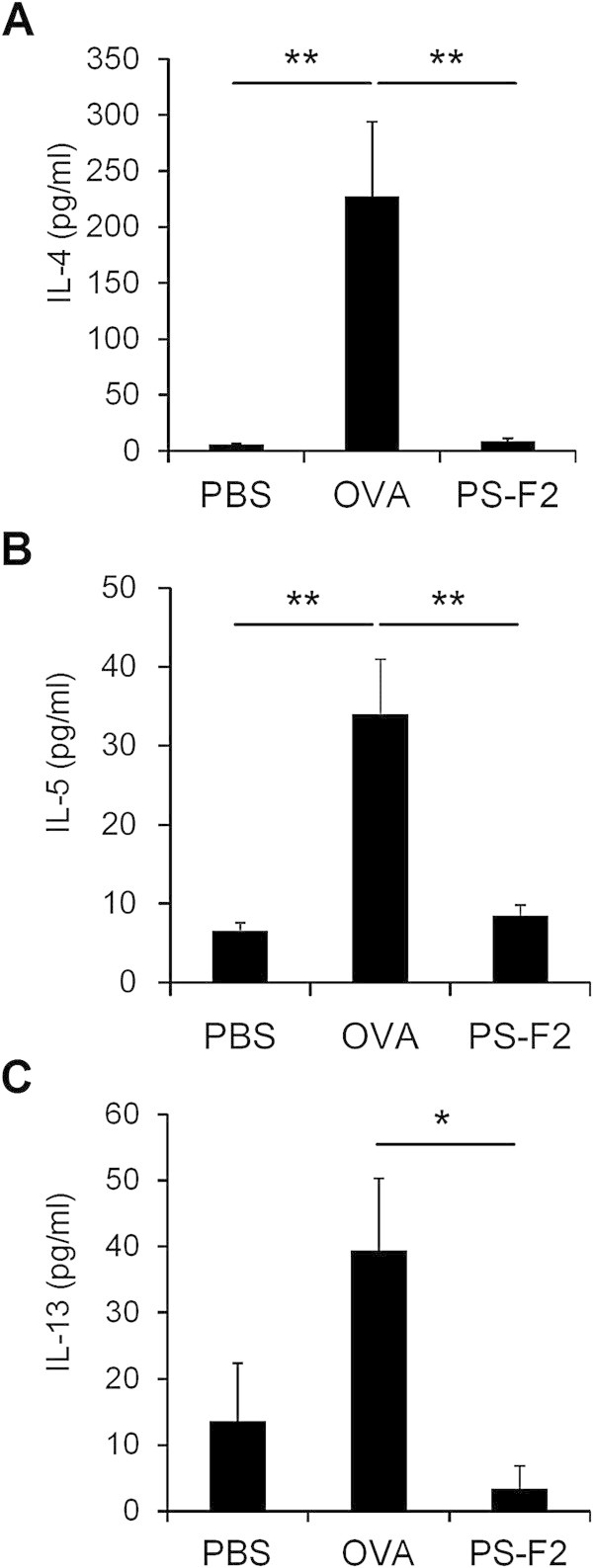
**PS-F2 administration suppresses OVA-induced Th2 cytokine production.** Mice were immunized, treated, and challenged as described in Figure 1. On day 28, BALF was collected, and levels of IL-4 **(A)**, IL-5 **(B)**, and IL-13 **(C)** were determined by ELISA (*n* = 10). Data shown are representative of 2 experiments. * *P* < 0.05, ** *P* < 0.01.

**Figure 6:**
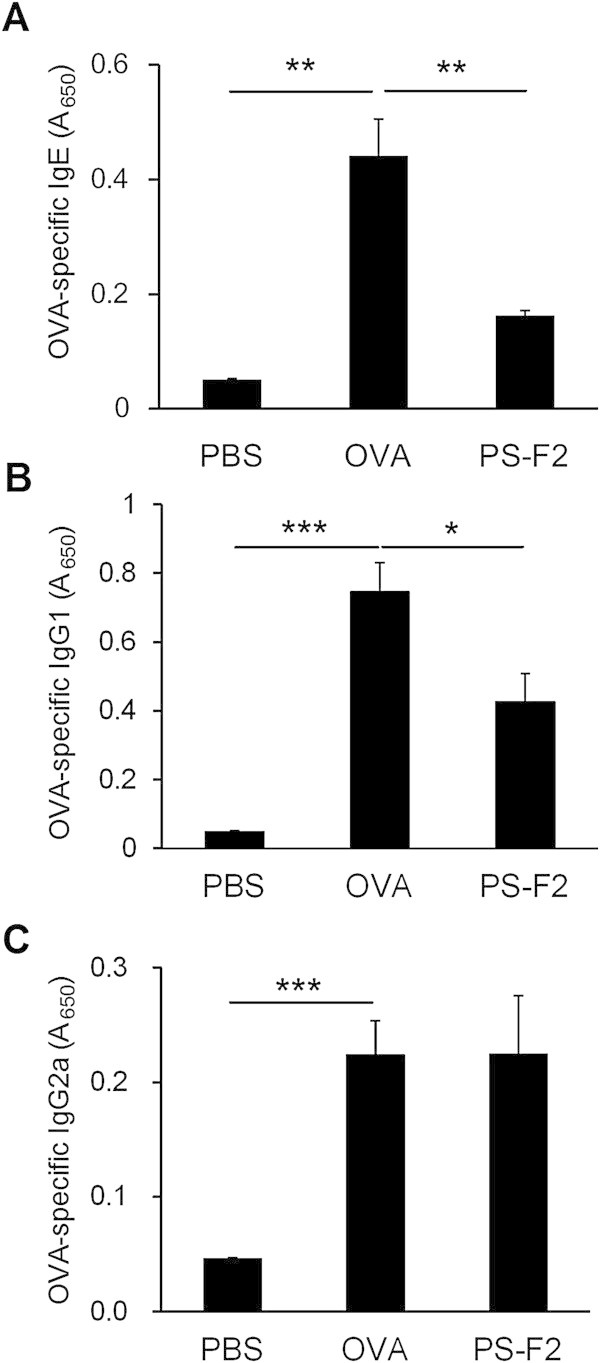
**Effect of PS-F2 administration on the production of OVA-specific antibodies.** Mice were immunized, treated, and challenged as described in Figure 1. **(A)** On day 14, serum levels of OVA-specific IgE were determined by ELISA. **(B, C)** On day 28, serum levels of OVA-specific IgG1 **(B)**, and IgG2a **(C)** were determined by ELISA. *n* = 10 in all groups. Data shown are representative of 2 experiments. * *P* < 0.05, ** *P* < 0.01, *** *P* < 0.001.

The cytokines produced by allergen-specific Th2 lymphocytes are thought to be responsible for many symptoms of asthma. To investigate whether PS-F2 treatment attenuated OVA-induced AHR and inflammation by suppressing the development of a Th2 immune response, we first measured levels of IL-4, IL-5, and IL-13 (protypical Th2 cytokines) in BALF. As expected, mice sensitized and challenged with OVA produced significant amounts of IL-4 (Figure 5A), IL-5 (Figure 5B), and IL-13 (Figure 5C), and in contrast, levels of these cytokines were markedly reduced in PS-F2-treated animals (Figure 5 and Additional file 3). We further analyzed the production of OVA-specific IgE, IgG1, and IgG2a in serum; the former two isotypes are indicators of Th2-skewed inflammation and IgG2a is a marker of Th1-skewed inflammation. As shown in Figure 6 and Additional file 4, OVA immunization and challenge induced a significant production of OVA-specific IgE (Figure 6A), IgG1 (Figure 6B), and IgG2a (Figure 6C) antibodies. PS-F2 treatment resulted in significant reduction in OVA-specific IgE (Figure 6A) and IgG1 (Figure 6B) levels; while the production of OVA-specific IgG2a was less affected (Figure 6C), indicating that PS-F2 did not induce a general suppression in antibody production. Together these data showed that PS-F2 treatment could effectively prevent the development of a Th2-skewed inflammation in OVA-sensitized/challenged mice.

## Discussion

In a previous study, we found that PS-F2, the polysaccharides produced by *G. formosanum*, could function as an adjuvant and prime an antigen-specific Th1 immune response (Pi et al. 2014). This observation prompted us to investigate whether PS-F2 treatment could attenuate Th2-mediated immunopathology *in vivo* through modulating the Th1/Th2 balance. In this study, we tested our hypothesis using an OVA-induced allergic asthma model in mice. Our data showed that administration of PS-F2 to OVA-sensitized/challenged animals attenuated all features of allergic asthma, including bronchial inflammation, the development of AHR, the secretion of Th2 cytokines, and the production of OVA-specific IgE and IgG1 antibodies. PS-F2 is therefore a novel agent that can be used to prevent allergic asthma. These findings also provide a new insight into the immunomodulatory functions of the medicinal fungus *Ganoderma*.

The major finding in this report is that PS-F2 treatment significantly lowered the degree of AHR and airway inflammation in OVA-sensitized/challenged mice. In this widely used murine asthma model, animals are first sensitized systemically by repeated immunization with OVA + alum, which induces a strong Th2 immune response that is associated with the production of OVA-specific IgE and IgG1 (Beck and Spiegelberg 1989). Airway challenge of presensitized mice with OVA then induces airway inflammation with preferential eosinophilic infiltration and AHR (Zhang et al. 1997). Although this model does not cause chronic airway inflammation and remodeling, as seen in human asthma, it does mimic the allergic pulmonary inflammation and AHR seen in humans and is therefore useful for evaluating the potential therapeutic agents of asthma (Szelenyi 2000). Our data show that PS-F2 treatment effectively suppressed the development of both AHR and airway inflammation, suggesting that these two events are closely associated. Indeed, airway allergic inflammation is thought to be the basis for AHR, and both cellular and noncellular aspects of airway inflammation are hypothesized to be important (Wills-Karp 1999).

In the OVA-sensitized/challenged mice, we detected marked infiltration of eosinophils, monocytes, and lymphocytes into the lungs, which are characteristics of late-phase responses in allergic asthma (Wills-Karp 1999). Among these cells, eosinophils are thought to be the key effector cells contributing to airway dysfunction and tissue remodeling in allergic asthma (Jacobsen et al. 2007). The reduced eosinophilia in the lungs of PS-F2-treated animals was most likely due to the reduced level of IL-5. T lymphocytes, in particular CD4^+^ T cells, function as orchestrators of the inflammatory response and play an important role in the pathogenesis of asthma (Wills-Karp 1999). In PS-F2-treated mice, a reduced number of lymphocytes was associated with reduced levels of IL-4, IL-5, and IL-13 in BALF, indicating that in OVA-sensitized/challenged mice, the majority of lymphocytes recruited to the lungs were Th2 cytokine-producing cells. Together these data support our hypothesis that administration of PS-F2, which is a Th1 adjuvant, before allergen exposure may suppress the induction of a Th2-mediated allergic inflammation, and therefore attenuates airway pathology and AHR.

The development of Th1/Th2/Th17 immune responses is regulated by the signals provided by APCs, in particular the cytokines produced by APCs upon activation (Zhu et al. 2010). It is therefore possible to treat allergic disorders with adjuvants that can skew the T cell response from Th2 to Th1. Based on this assumption, synthetic TLR agonists have been developed and tested clinically to treat asthma and allergies (Bezemer et al. 2012). CpG oligodeoxynucleotides (ODNs), which activate TLR9, are the most extensively investigated synthetic TLR agonists in preclinical and clinical studies for the treatment of allergic asthma (Fonseca and Kline 2009). It was reported that CpG ODNs induce the production of interferon (IFN)-γ, IL-6, and IL-12 by NK cells, B cells, and CD4^+^ T lymphocytes both *in vivo* and *in vitro* (Klinman et al. 1996). CpG ODN was also found to stimulate dendritic cells (DCs) to produce IL-12 and IL-10, which resulted in both a Th1 response and the induction of IL-10-producing regulatory T (Treg) cell production (Jarnicki et al. 2008). We found that PS-F2 could stimulate DCs to produce TNF-α, IL-12 p40, IL-6, and IL-10 (Pi et al. 2014), similar to the cytokines secreted by CpG-ODN-treated DCs. Therefore, in addition to the induction of a Th1 response, PS-F2 could possibly also induce the generation of Treg cells, and these responses may work together to confer the observed protective effects on allergic inflammation. The induction of indoleamine 2,3 dioxygenase (IDO) production has also been linked to the antiasthmatic effects of CpG ODNs (Hayashi et al. 2004); whether PS-F2 could induce IDO is a valid question and will require further investigation. Besides suppressing the development of allergic asthma, CpG ODNs have also been investigated as therapeutic agents for other allergic disorders, such as allergic rhinitis and conjunctivitis (Hussain et al. 2002; Magone et al. 2000; Rhee et al. 2004). The data obtained in this study also warrant future investigation of the use of PS-F2 in the treatment of other atopic diseases. In this study, PS-F2 was given alone to mice before and during the course of allergen exposure, and it will be worth testing the efficacy of other treatment protocols. For example, PS-F2 can be administered after the induction of disease to examine its therapeutic effect; PS-F2 can also be administered in conjunction with allergens and serve as an adjuvant in subcutaneous immunotherapy (SCIT) and sublingual immunotherapy (SLIT).

Natural products have been used as major sources of medicine throughout the world for centuries, and many of them exhibit immunomodulatory functions (Chlubnova et al. 2011; Ramberg et al. 2010). Because allergic asthma is a Th2-mediated inflammatory disease, theoretically, substances that exhibit antiinflammatory and/or Th1-skewing properties could potentially be used to ameliorate the disease. Numerous herbs, phytochemicals, and vitamins have been investigated for their interactions with the immune system and used as complementary and alternative medicines in treating atopic disorders (Chang et al. 2013; Chang et al. 2011; Hwang et al. 2012; Lee et al. 2012a; Lee et al. 2010; Lee et al. 2012b; Mainardi et al. 2009; Rao et al. 2013; Wang et al. 2013). *Ganoderma* is a medicinal fungus that is considered to be a therapeutic biofactory with numerous pharmacologically active components (Paterson 2006). In this study, we provide evidence that PS-F2, the extracellular polysaccharides produced during the submerged culture of *G. formosanum*, effectively suppress the development of allergic asthma in mice. Similar to our observation, Liu et al. reported that polysaccharides extracted from another medicinal fungus, *Antrodia camphorate*, could stimulate the production of IL-12 and IL-10 in DCs and alleviate OVA-induced allergic asthma in mice (Liu et al. 2010). Jan et al. found that polysaccharides extracted from the fruiting bodies of *G. lucidum* could stimulate the production of IFN-γ and downregulate IL-5 production from T cells co-cultured with DCs derived from asthmatic children (Jan et al. 2011). Therefore we speculate that other fungal polysaccharides with similar activity on DCs and/or with Th1 adjuvant activity may also exhibit antiallergic functions. Besides polysaccharides, a triterpenoid-rich extract of *G. tsugae* was also shown to attenuate the Th2 inflammation in a murine asthma model (Chen and Lin 2007). Therapeutic benefits on allergen-induced airway inflammation were observed when crude extracts of *G. lucidum* (Liu et al. 2003) and *G. tsugae* (Lin et al. 2006) were orally administered to mice. Whether PS-F2 maintains its antiasthmatic functions when given orally will require further investigation. Additional long-term experiments will also be needed to evaluate whether the preventive/therapeutic benefit of PS-F2 can be sustained and whether continuous administration of PS-F2 has any adverse effect.

## Conclusion

In conclusion, our data demonstrate that systemic administration of PS-F2 can suppress Th2-mediated bronchial inflammation and the development of AHR in a murine model of allergic asthma. Although the animal model used in this study may not fully recapitulate the human condition, our data suggest that PS-F2 has a potential to be developed into a preventive agent for allergic asthma.

## Materials and methods

### Animals

Female BALB/c mice (6 weeks old; average weight 20 g) were purchased from the National Laboratory Animal Center (Taipei, Taiwan). This study was carried out in strict accordance with the recommendations in the Guide for the Care and Use of Laboratory Animals of the Council of Agriculture, Taiwan. The protocol was approved by the Institute Animal Care and Use Committee of National Taiwan University, and all mice were kept in the animal facilities of the College of Life Science at National Taiwan University.

### PS-F2 and reagents

The major polysaccharide fraction PS-F2 was purified from the submerged culture of *G. formosanum* Chang et Chen (ATCC 76538) as previously described (Wang et al. 2011). The purified PS-F2 was passed through an endotoxin removal column (Detoxi-Gel Endotoxin Removing Gel, Thermo Scientific, Rockford, IL) and the endotoxin level in the samples was determined to be < 0.3 EU/mg by the Pyrotell Limulus Amebocytes Lysate (LAL) test (Associates of Cape Cod, Falmouth, MA). Chicken ovalbumin (OVA) and pentobarbital sodium were purchased from Sigma-Aldrich (St. Louis, MO) and passed though the Detoxi-Gel Endotoxin Removing Gel before use. Hanks’ balanced salt solution (HBSS) was purchased from Thermo Scientific HyClone (Logan, UT). Fetal bovine serum (FBS) was purchased from Biological Industries (Beit-Haemek, Israel). Dulbecco's phosphate buffered saline (DPBS) was purchased from Life Technologies (Gaithersburg, MD). All other chemicals were purchased from commercial sources at the highest purity available.

### Mice sensitization, challenge, and treatment

Female BALB/c mice were divided into three groups (PBS, OVA, and PS-F2) and treated as illustrated in Figure 1. In brief, mice were sensitized by intraperitoneal (i.p.) immunization with 50 μg of OVA emulsified in 4 mg of aluminum hydroxide (alum, Thermo Scientific) in a total volume of 200 μl in DPBS (OVA and PS-F2 groups) or DPBS alone (PBS group) on days 0, 10 and 20. To investigate the effect of PS-F2 on asthma induction, mice were treated i.p. with PBS (PBS and OVA groups) or 50 mg/kg of PS-F2 (PS-F2 group) on days −3, −1, 7, 9, 17, 19, 25, 26, and 27. Mice were challenged by intranasal (i.n.) injection of 100 μg of OVA on day 27 in all three groups. Twenty-four hours after OVA challenge, AHR was measured, and blood was collected for measuring OVA-specific antibodies. Mice were then sacrificed, the bronchoalveolar lavage fluid (BALF) was harvested, and lung sections were prepared.

### OVA-specific antibody analysis

OVA-specific IgE in serum was measured by ELISA on day 14 after OVA immunization. OVA-specific IgG1 and IgG2a in serum were measured by ELISA on day 28 after OVA immunization. Ninety-six-well plates were coated with 10 μg/ml OVA. After overnight incubation at 4°C, plates were washed with PBS containing 0.05% Tween 20 (PBST) and blocked with 1% bovine serum albumin in PBS for 2 h at room temperature. Serum samples were diluted and added to each well overnight at 4°C. The plates were then washed with PBST, and biotin-conjugated anti-mouse IgE (1:100), IgG1 (1:10000), and IgG2a (1:1000) (BD Biosciences, San Jose, CA) were added for 2 h at room temperature. Streptavidin-conjugated horseradish peroxidase was added for another 30 min at room temperature. Finally, the reaction was developed by H_2_O_2_ and tetramethylbenzidine (BD Biosciences), and 650 nm absorbance was measured using a microplate reader (Thermo Scientific).

### Measurement of airway hyperresponsiveness (AHR)

At 24 h after i.n. challenge of OVA, airway hyperresponsiveness was assessed by invasive measurement of lung resistance and dynamic compliance. In brief, mice were anesthetized with 80 mg/kg pentobarbital sodium, tracheostomized, and mechanically ventilated at a rate of 150 breaths/min and a tidal volume of 0.3 ml/kg with a computer-controlled small animal ventilator and pulmonary function analyzer (flexiVent, SCIREQ, Montreal, PQ, Canada), which was used for measuring respiratory mechanics and lung function through forced oscillation. To induce the symptoms of bronchial contraction and AHR, mice were exposed to aerosolized PBS and methacholine for 20 sec. The pressure and volume change of flow were recorded by electronic differentiation, and lung resistance (R_L_) was calculated automatically by the flexiVent software. The ratio of R_L_ was measured after PBS nebulization with increasing doses of methacholine (1.56, 3.13, 6.25, 12.5, 25 mg/ml).

### Bronchoalveolar lavage and BALF analysis

Bronchoalveolar lavage was performed by instilling 1 ml of HBSS containing 2% FBS to the trachea twice via a trachea cannula (Angiocatch®, BD Biosciences), and BALF was harvested by gentle aspiration. After centrifugation (300 × g, 3 min), BALF supernatants were assayed for IL-4, IL-5, and IL-13 by ELISA (eBioscience, San Diego, CA). BALF cells resuspended in HBSS (1 × 10^5^ cells/ml) were cytospined (300 × g, 5 min) onto slides and stained with Liu’s staining. Differential cell counts were performed under a microscope, and a minimum of 300 cells were counted and classified into eosinophils, monocytes, lymphocytes, and neutrophils based on the standard morphological criteria.

### Lung histology

After sacrifice, the lungs of mice were immediately removed and fixed with 10% neutral phosphate-buffered formalin. The lung tissues were embedded in paraffin and cut into 5-μm-thick sections. The sections were then stained with hematoxylin and eosin and examined under a light microscope.

### Statistical analysis

Statistical analysis was performed using an unpaired, two-tailed Student's *t*-test and a *P* < 0.05 was considered significant. Data are reported as mean ± SEM.

## Electronic supplementary material

Additional file 1: **Effect of PS-F2 treatment on OVA-induced AHR in mice.** Mice were immunized, treated, and challenged as described in Figure 1. AHR (R_L_ ratio) was measured as described in Figure 2. (PDF 12 KB)

Additional file 2: **Effect of PS-F2 treatment on bronchial inflammation in OVA-challenged mice.** Mice were immunized, treated, and challenged as described in Figure 1. On day 28, the numbers of total BALF cells and inflammatory cells were determined as described in Figure 3. (PDF 14 KB)

Additional file 3: **Effect of PS-F2 treatment on OVA-induced Th2 cytokine production.** Mice were immunized, treated, and challenged as described in Figure 1. On day 28, levels of Th2 cytokines in BALF were determined as described in Figure 5. (PDF 10 KB)

Additional file 4: **Effect of PS-F2 treatment on the production of OVA-specific antibodies.** Mice were immunized, treated, and challenged as described in Figure 1. On day 28, serum levels of OVA-specific antibodies were determined as described in Figure 6. (PDF 11 KB)

## Abbreviations

AHR: Airway hyperresponsiveness
Alum: Aluminum hydroxide
APCs: Antigen-presenting cells
BALF: Bronchoalveolar lavage fluid
DCs: Dendritic cells
DPBS: Dulbecco's phosphate buffered saline
FBS: Fetal bovine serum
HBSS: Hanks’ balanced salt solution
IDO: Indoleamine 2,3 dioxygenase
IFN: Interferon
IL: Interleukin
R_L_: Lung resistance
OVA: Ovalbumin
ODNs: Oligodeoxynucleotides
Th: T helper
TLR4: Toll-like receptor 4
Treg: Regulatory T.

## References

[CR1] Barnes PJ (2004). New drugs for asthma. Nat Rev Drug Discov.

[CR2] Beck L, Spiegelberg HL (1989). The polyclonal and antigen-specific IgE and IgG subclass response of mice injected with ovalbumin in alum or complete Freund's adjuvant. Cell Immunol.

[CR3] Bezemer GF, Sagar S, van Bergenhenegouwen J, Georgiou NA, Garssen J, Kraneveld AD, Folkerts G (2012). Dual role of Toll-like receptors in asthma and chronic obstructive pulmonary disease. Pharmacol Rev.

[CR4] Boh B, Berovic M, Zhang J, Zhi-Bin L (2007). *Ganoderma lucidum* and its pharmaceutically active compounds. Biotechnol Annu Rev.

[CR5] Burks AW, Calderon MA, Casale T, Cox L, Demoly P, Jutel M, Nelson H, Akdis CA (2013). Update on allergy immunotherapy: American Academy of Allergy, Asthma & Immunology/European Academy of Allergy and Clinical Immunology/PRACTALL consensus report. J Allergy Clin Immunol.

[CR6] Chang HC, Gong CC, Chan CL, Mak OT (2013). A nebulized complex traditional Chinese medicine inhibits Histamine and IL-4 production by ovalbumin in guinea pigs and can stabilize mast cells in vitro. BMC Complement Altern Med.

[CR7] Chang HC, Gong CC, Chen JL, Mak OT (2011). Inhibitory effects of inhaled complex traditional Chinese medicine on early and late asthmatic responses induced by ovalbumin in sensitized guinea pigs. BMC Complement Altern Med.

[CR8] Chen ML, Lin BF (2007). Effects of triterpenoid-rich extracts of *Ganoderma tsugae* on airway hyperreactivity and Th2 responses in vivo. Int Arch Allergy Immunol.

[CR9] Chlubnova I, Sylla B, Nugier-Chauvin C, Daniellou R, Legentil L, Kralova B, Ferrieres V (2011). Natural glycans and glycoconjugates as immunomodulating agents. Nat Prod Rep.

[CR10] Fitzhugh DJ, Lockey RF (2011). Allergen immunotherapy: a history of the first 100 years. Curr Opin Allergy Clin Immunol.

[CR11] Fonseca DE, Kline JN (2009). Use of CpG oligonucleotides in treatment of asthma and allergic disease. Adv Drug Deliv Rev.

[CR12] Galli SJ, Tsai M, Piliponsky AM (2008). The development of allergic inflammation. Nature.

[CR13] Hamid Q, Tulic M (2009). Immunobiology of asthma. Annu Rev Physiol.

[CR14] Hayashi T, Beck L, Rossetto C, Gong X, Takikawa O, Takabayashi K, Broide DH, Carson DA, Raz E (2004). Inhibition of experimental asthma by indoleamine 2,3-dioxygenase. J Clin Invest.

[CR15] Hussain I, Jain VV, Kitagaki K, Businga TR, O'Shaughnessy P, Kline JN (2002). Modulation of murine allergic rhinosinusitis by CpG oligodeoxynucleotides. Laryngoscope.

[CR16] Hwang JS, Kwon HK, Kim JE, Rho J, Im SH (2012). Immunomodulatory effect of water soluble extract separated from mycelium of Phellinus linteus on experimental atopic dermatitis. BMC Complement Altern Med.

[CR17] Ingram JL, Kraft M (2012). IL-13 in asthma and allergic disease: asthma phenotypes and targeted therapies. J Allergy Clin Immunol.

[CR18] Jacobsen EA, Ochkur SI, Lee NA, Lee JJ (2007). Eosinophils and asthma. Curr Allergy Asthma Rep.

[CR19] Jan RH, Lin TY, Hsu YC, Lee SS, Lo SY, Chang M, Chen LK, Lin YL (2011). Immuno-modulatory activity of *Ganoderma lucidum*-derived polysacharide on human monocytoid dendritic cells pulsed with Der p 1 allergen. BMC Immunol.

[CR20] Jarnicki AG, Conroy H, Brereton C, Donnelly G, Toomey D, Walsh K, Sweeney C, Leavy O, Fletcher J, Lavelle EC, Dunne P, Mills KH (2008). Attenuating regulatory T cell induction by TLR agonists through inhibition of p38 MAPK signaling in dendritic cells enhances their efficacy as vaccine adjuvants and cancer immunotherapeutics. J Immunol.

[CR21] Klinman DM, Yi AK, Beaucage SL, Conover J, Krieg AM (1996). CpG motifs present in bacteria DNA rapidly induce lymphocytes to secrete interleukin 6, interleukin 12, and interferon gamma. Proc Natl Acad Sci U S A.

[CR22] Lee CC, Wang CN, Kang JJ, Liao JW, Chiang BL, Chen HC, Hu CM, Lin CD, Huang SH, Lai YT (2012). Antiallergic asthma properties of brazilin through inhibition of TH2 responses in T cells and in a murine model of asthma. J Agric Food Chem.

[CR23] Lee CC, Wang CN, Lai YT, Kang JJ, Liao JW, Chiang BL, Chen HC, Cheng YW (2010). Shikonin inhibits maturation of bone marrow-derived dendritic cells and suppresses allergic airway inflammation in a murine model of asthma. Br J Pharmacol.

[CR24] Lee MY, Shin IS, Lim HS, Shin HK (2012). A water extract of Samchulkunbi-tang attenuates airway inflammation by inhibiting inos and MMP-9 activities in an ovalbumin-induced murine asthma model. BMC Complement Altern Med.

[CR25] Leikauf GD (2002). Hazardous air pollutants and asthma. Environ Health Perspect.

[CR26] Li QZ, Wang XF, Zhou XW (2011). Recent status and prospects of the fungal immunomodulatory protein family. Crit Rev Biotechnol.

[CR27] Lin JY, Chen ML, Chiang BL, Lin BF (2006). *Ganoderma tsugae* supplementation alleviates bronchoalveolar inflammation in an airway sensitization and challenge mouse model. Int Immunopharmacol.

[CR28] Liu KJ, Leu SJ, Su CH, Chiang BL, Chen YL, Lee YL (2010). Administration of polysaccharides from Antrodia camphorata modulates dendritic cell function and alleviates allergen-induced T helper type 2 responses in a mouse model of asthma. Immunology.

[CR29] Liu YH, Tsai CF, Kao MC, Lai YL, Tsai JJ (2003). Effectiveness of Dp2 nasal therapy for Dp2- induced airway inflammation in mice: using oral *Ganoderma lucidum* as an immunomodulator. J Microbiol Immunol Infect.

[CR30] Magone MT, Chan CC, Beck L, Whitcup SM, Raz E (2000). Systemic or mucosal administration of immunostimulatory DNA inhibits early and late phases of murine allergic conjunctivitis. Eur J Immunol.

[CR31] Mainardi T, Kapoor S, Bielory L (2009). Complementary and alternative medicine: herbs, phytochemicals and vitamins and their immunologic effects. J Allergy Clin Immunol.

[CR32] Paterson RR (2006). *Ganoderma* - a therapeutic fungal biofactory. Phytochemistry.

[CR33] Pelaia G, Vatrella A, Maselli R (2012). The potential of biologics for the treatment of asthma. Nat Rev Drug Discov.

[CR34] Pi CC, Chu CL, Lu CY, Zhuang YJ, Wang CL, Yu YH, Wang HY, Lin CC, Chen CJ (2014). Polysaccharides from Ganoderma formosanum function as a Th1 adjuvant and stimulate cytotoxic T cell response in vivo. Vaccine.

[CR35] Ramberg JE, Nelson ED, Sinnott RA (2010). Immunomodulatory dietary polysaccharides: a systematic review of the literature. Nutr J.

[CR36] Rao YK, Chen YC, Fang SH, Lai CH, Geethangili M, Lee CC, Tzeng YM (2013). Ovatodiolide inhibits the maturation of allergen-induced bone marrow-derived dendritic cells and induction of Th2 cell differentiation. Int Immunopharmacol.

[CR37] Rhee CS, Libet L, Chisholm D, Takabayashi K, Baird S, Bigby TD, Lee CH, Horner AA, Raz E (2004). Allergen-independent immunostimulatory sequence oligodeoxynucleotide therapy attenuates experimental allergic rhinitis. Immunology.

[CR38] Rodrigo GJ, Neffen H, Castro-Rodriguez JA (2011). Efficacy and safety of subcutaneous omalizumab vs placebo as add-on therapy to corticosteroids for children and adults with asthma: a systematic review. Chest.

[CR39] Szelenyi I (2000). Animal models of bronchial asthma. Inflamm Res.

[CR40] Walsh GM (2013). Profile of reslizumab in eosinophilic disease and its potential in the treatment of poorly controlled eosinophilic asthma. Biologics.

[CR41] Wang CC, Lin YR, Liao MH, Jan TR (2013). Oral supplementation with areca-derived polyphenols attenuates food allergic responses in ovalbumin-sensitized mice. BMC Complement Altern Med.

[CR42] Wang CL, Lu CY, Pi CC, Zhuang YJ, Chu CL, Liu WH, Chen CJ (2012). Extracellular polysaccharides produced by *Ganoderma formosanum* stimulate macrophage activation via multiple pattern-recognition receptors. BMC Complement Altern Med.

[CR43] Wang CL, Pi CC, Kuo CW, Zhuang YJ, Khoo KH, Liu WH, Chen CJ (2011). Polysaccharides purified from the submerged culture of *Ganoderma formosanum* stimulate macrophage activation and protect mice against *Listeria monocytogenes* infection. Biotechnol Lett.

[CR44] Wasser SP (2002). Medicinal mushrooms as a source of antitumor and immunomodulating polysaccharides. Appl Microbiol Biotechnol.

[CR45] Wenzel S, Wilbraham D, Fuller R, Getz EB, Longphre M (2007). Effect of an interleukin-4 variant on late phase asthmatic response to allergen challenge in asthmatic patients: results of two phase 2a studies. Lancet.

[CR46] Wills-Karp M (1999). Immunologic basis of antigen-induced airway hyperresponsiveness. Annu Rev Immunol.

[CR47] Xu Z, Chen X, Zhong Z, Chen L, Wang Y (2011). *Ganoderma lucidum* polysaccharides: immunomodulation and potential anti-tumor activities. Am J Chin Med.

[CR48] Zhang Y, Lamm WJ, Albert RK, Chi EY, Henderson WR, Lewis DB (1997). Influence of the route of allergen administration and genetic background on the murine allergic pulmonary response. Am J Respir Crit Care Med.

[CR49] Zhu J, Yamane H, Paul WE (2010). Differentiation of effector CD4 T cell populations (*). Annu Rev Immunol.

