# Supplementary material for: Ganoderma formosanum polysaccharides attenuate Th2 inflammation and airway hyperresponsiveness in a murine model of allergic asthma
Source: Springerplus. 2014 Jun 12;3:297. doi: 10.1186/2193-1801-3-297 (PMC4072879; doi:10.1186/2193-1801-3-297)
Supplement: Supplementary file 1 — Additional file 1: Effect of PS-F2 treatment on OVA-induced AHR in mice. Mice were immunized, treated, and challenged as described in Figure 1. AHR (RL ratio) was measured as described in Figure 2. (PDF 12 KB) [file 40064_2014_1008_MOESM1_ESM.pdf]

Additional file 1

Effect of PS-F2 treatment on OVA-induced AHR in mice. Mice were immunized, treated, and challenged as described in Figure 1. AHR (R_L ratio) was measured as described in Figure 2.

	R_L ratio					
	Methacholine (mg/ml)					
	0	1.56	3.13	6.25	12.5	25
PBS	1	1.01 ± 0.06	1.17 ± 0.11	1.87 ± 0.25	2.45 ± 0.31	2.63 ± 0.58*
OVA	1	1.12 ± 0.03	1.52 ± 0.14	2.52 ± 0.48	4.17 ± 0.76	5.71 ± 1.04
PS-F2	1	1.23 ± 0.15	1.38 ± 0.09	1.98 ± 0.24	2.80 ± 0.35*	2.99 ± 0.34*

Data are reported as mean ± SEM (n = 10). *$P < 0.05$ vs. OVA group in the same column.
