# Supplementary material for: Ganoderma formosanum polysaccharides attenuate Th2 inflammation and airway hyperresponsiveness in a murine model of allergic asthma
Source: Springerplus. 2014 Jun 12;3:297. doi: 10.1186/2193-1801-3-297 (PMC4072879; doi:10.1186/2193-1801-3-297)
Supplement: Supplementary file 2 — Additional file 2: Effect of PS-F2 treatment on bronchial inflammation in OVA-challenged mice. Mice were immunized, treated, and challenged as described in Figure 1. On day 28, the numbers of total BALF cells and inflammatory cells were determined as described in Figure 3. (PDF 14 KB) [file 40064_2014_1008_MOESM2_ESM.pdf]

Additional file 2

Effect of PS-F2 treatment on bronchial inflammation in OVA-challenged mice. Mice were immunized, treated, and challenged as described in Figure 1. On day 28, the numbers of total BALF cells and inflammatory cells were determined as described in Figure 3.

	Numbers of inflammatory cells in BALF (× 10^5 cells)				
	Total leukocyte	Eosinophil	Monocyte	Lymphocyte	Neutrophil
PBS	2.50 ± 0.24***	0.36 ± 0.08**	1.53 ± 0.23**	0.46 ± 0.10	0.20 ± 0.06*
OVA	6.71 ± 0.94	1.18 ± 0.26	4.66 ± 1.02	1.34 ±0.48	0.47 ± 0.10
PS-F2	3.20 ± 0.46**	0.32 ± 0.10**	2.55 ± 0.51	0.27 ± 0.08*	0.04 ± 0.02**

Data are reported as mean ± SEM (n = 10). *P < 0.05, ** P < 0.01, *** P < 0.001 vs. OVA group in the same column.
