# Supplementary material for: Ganoderma formosanum polysaccharides attenuate Th2 inflammation and airway hyperresponsiveness in a murine model of allergic asthma
Source: Springerplus. 2014 Jun 12;3:297. doi: 10.1186/2193-1801-3-297 (PMC4072879; doi:10.1186/2193-1801-3-297)
Supplement: Supplementary file 3 — Additional file 3: Effect of PS-F2 treatment on OVA-induced Th2 cytokine production. Mice were immunized, treated, and challenged as described in Figure 1. On day 28, levels of Th2 cytokines in BALF were determined as described in Figure 5. (PDF 10 KB) [file 40064_2014_1008_MOESM3_ESM.pdf]

Additional file 3

Effect of PS-F2 treatment on OVA-induced Th2 cytokine production. Mice were immunized, treated, and challenged as described in Figure 1. On day 28, levels of Th2 cytokines in BALF were determined as described in Figure 5.

	IL-4 (pg/ml)	IL-5 (pg/ml)	IL-13 (pg/ml)
PBS	5.98 ± 0.74**	6.51 ± 1.04**	13.57 ± 8.82
OVA	227.28 ± 66.91	34.03 ± 6.93	39.35 ± 10.95
PS-F2	8.76 ± 2.61**	8.51 ± 1.32**	3.42 ± 3.43*

Data are reported as mean ± SEM (n = 10). *P < 0.05, ** P < 0.01 vs. OVA group in the same column.
