# Supplementary material for: Ganoderma formosanum polysaccharides attenuate Th2 inflammation and airway hyperresponsiveness in a murine model of allergic asthma
Source: Springerplus. 2014 Jun 12;3:297. doi: 10.1186/2193-1801-3-297 (PMC4072879; doi:10.1186/2193-1801-3-297)
Supplement: Supplementary file 4 — Additional file 4: Effect of PS-F2 treatment on the production of OVA-specific antibodies. Mice were immunized, treated, and challenged as described in Figure 1. On day 28, serum levels of OVA-specific antibodies were determined as described in Figure 6. (PDF 11 KB) [file 40064_2014_1008_MOESM4_ESM.pdf]

**Additional file 4**

**Effect of PS-F2 treatment on the production of OVA-specific antibodies.** Mice were immunized, treated, and challenged as described in Figure 1. On day 28, serum levels of OVA-specific antibodies were determined as described in Figure 6.

| | OVA-specific antibodies ($A_{650}$) | | |
|---|---|---|---|
| | IgE | IgG1 | IgG2a |
| PBS | 0.051 ± 0.001** | 0.048 ± 0.003*** | 0.046 ± 0.001*** |
| OVA | 0.442 ± 0.064 | 0.746 ± 0.085 | 0.224 ± 0.030 |
| PS-F2 | 0.162 ± 0.010** | 0.425 ± 0.082* | 0.224 ± 0.051 |

Data are reported as mean ± SEM ($n$ = 10). *$P$ < 0.05, ** $P$ < 0.01, *** $P$ < 0.001 vs. OVA group in the same column.
